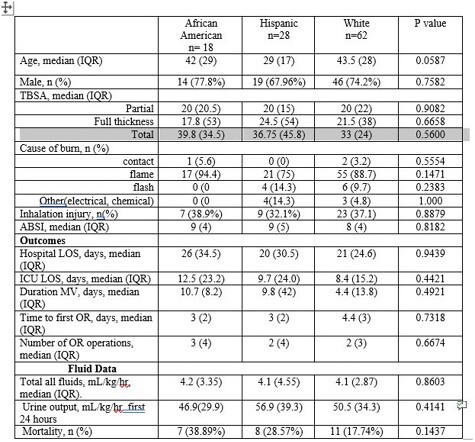# 702 Ethnicity Does Not Change Burn Resuscitation and Time to First Excision

**DOI:** 10.1093/jbcr/irae036.247

**Published:** 2024-04-17

**Authors:** Kareem R Abdelfattah, Rebecca Coffey, Janie J Faris, Audra Clark, Benjamin Levi

**Affiliations:** UT-Southwestern Medical Center, Dallas, TX; Parkland Health, Keller, TX; Parkland Hospital, Dallas, TX; University of Texas Southwestern, Dallas, TX; UTSW, Dallas, TX; UT-Southwestern Medical Center, Dallas, TX; Parkland Health, Keller, TX; Parkland Hospital, Dallas, TX; University of Texas Southwestern, Dallas, TX; UTSW, Dallas, TX; UT-Southwestern Medical Center, Dallas, TX; Parkland Health, Keller, TX; Parkland Hospital, Dallas, TX; University of Texas Southwestern, Dallas, TX; UTSW, Dallas, TX; UT-Southwestern Medical Center, Dallas, TX; Parkland Health, Keller, TX; Parkland Hospital, Dallas, TX; University of Texas Southwestern, Dallas, TX; UTSW, Dallas, TX; UT-Southwestern Medical Center, Dallas, TX; Parkland Health, Keller, TX; Parkland Hospital, Dallas, TX; University of Texas Southwestern, Dallas, TX; UTSW, Dallas, TX

## Abstract

**Introduction:**

Burn patients sustaining a greater than 20% total body surface area (TBSA) require a systematic approach for resuscitation as these patients have a large systemic inflammatory response causing large fluid shifts. Ethnicity has been shown to influence overall clinical outcome from burn injury, but differences in the initial resuscitation between ethnicities has not been explored. This study was to identify potential racial disparities that might exist during the initial resuscitation and time to first excision.

**Methods:**

A convenience sample of all burn patients 14 years or older admitted to one burn center between 1/1/2020 and 12/31/2021 and require formal fluid resuscitation were included. Baseline demographics, burn data, past medical history, inhalation injury and mechanical ventilation (MV) were collected. Outcomes included hospital and ICU length of stay (LOS), duration of MV, total IV fluids (ml/kg/hr.), urine output (ml/kg/hr.), and hospital mortality. Nominal data was analyzed using fishers exact and Kruskal Wallis for continuous variables. A p value of < 0.05 was considered significant.

**Results:**

A total of 109 subjects were included in analysis. See table 1

**Conclusions:**

Burn outcomes have been shown to be influenced by ethnicity, although the causes of these inequities are unknown. This is the first study to evaluate the potential impact of multiple ethnicities on initial burn fluid resuscitation and time to first excision. No statistically significant difference in baseline demographics, LOS, duration of MV or overall hospital mortality were found. There appeared to be no disparities in burn resuscitation and time to first excision based on ethnicity, however further studies are needed.

**Applicability of Research to Practice:**

Effects of health disparities on burn treatment can affect outcomes